# Accumulation of CD5^+^CD19^+^ B lymphocytes expressing PD-1 and PD-1L in hypertrophied pharyngeal tonsils

**DOI:** 10.1007/s10238-015-0385-y

**Published:** 2015-08-29

**Authors:** Paulina Wlasiuk, Artur Niedzielski, Katarzyna Skorka, Agnieszka Karczmarczyk, Joanna Zaleska, Malgorzata Zajac, Maciej Putowski, Elzbieta Pac-Kozuchowska, Krzysztof Giannopoulos

**Affiliations:** 1 Experimental Hematooncology Department, Medical University of Lublin, Chodzki 4a, 20093 Lublin, Poland; 2 Otoneurology Laboratory, Medical University of Lublin, Chodzki 2, 20093 Lublin, Poland; 3 Department of Paediatrics, Medical University of Lublin, Chodzki 2, 20093 Lublin, Poland

**Keywords:** PD-1, PD-1L, CD5^+^CD19^+^ B cells, Tonsillitis, Chronic antigenic stimulation

## Abstract

Programmed death-1 (PD-1) is one of the most important inhibitory co-receptors expressed predominantly on activated T and B lymphocytes whose expression could be sustained by permanent antigenic stimulation accompanying chronic or recurrent tonsillitis. The expression of PD-1 and PD-1L was analyzed using flow cytometry on hypertrophied tonsils collected from 57 children. We observed high expression of PD-1 and PD-1L on certain lymphocytes subpopulations of hypertrophied tonsils; among T cells, the expression of PD-1 on protein level was higher on CD4^+^ cells (70.3 %) than on CD8^+^ cells (35 %). Interestingly, a limited expression of PD-1 was observed on CD19^+^ B lymphocytes (6.5 %), while CD5^+^CD19^+^ B cells overexpressed PD-1 (52.5 %). Moreover, the expression of PD-1L was also higher on CD5^+^CD19^+^ B cells (16.5 %) than on CD19^+^ B cells (3.5 %) and on CD4^+^ T cells (20 %) than on CD8^+^ T cells (10 %). PD-1 and PD-1L expressions correlated only on CD5^+^CD19^+^ cells. In conclusion, high expression of PD-1 and PD-1L on T and B cells could represent hallmark of immune system adaptation to chronic antigenic exposition in patients with tonsillitis.

## Introduction

Recurrent viral and bacterial infections are the major underlying causes of tonsillitis, which typically is a self-limiting localized inflammation of the oropharynx. These infections upon continuous antigenic stimulation could induce histomorphological and functional changes in the tonsils, making tonsillectomy necessary [1]. The tonsils play a key role in host defense against invading antigens of the upper respiratory tract being a site of residence of the intraepithelial immunoglobulin-producing B cells that represent approximately of 50 % cells [2]. A smaller percentage of tonsillar lymphocytes are T cells with a greater number of CD4 than CD8 cells, the former predominantly localized in clusters with B cells. In subepithelial area of tonsils, B1 lymphocytes represent about 15 % of B cells. The subpopulation of B1 cells, B1a, characterized by surface co-expression of CD5 and CD19 predominate in fetal life, and their frequency decreases with age. Interestingly, it has been observed that frequency of B1a cells could increase again in elderly [3]. The B1a lymphocytes are found in lymph nodes, spleen, extranodal tissue and tonsils. The main role of B1a lymphocytes is to produce “natural,” low-affinity and polyreactive IgM which could bind different bacterial antigens and autoantigens representing a bridge between innate and adaptive immune responses [3, 4].

The main purpose of this work was to characterize the expression of programmed death-1 (PD-1) and programmed death-1 ligand (PD-1L) on certain lymphocytes subpopulations of hypertrophied tonsils obtained by tonsillectomy from children with recurrent exacerbations of chronic tonsillar infections. The expression of PD-1 has been described as a marker of cell exhaustion in chronic inflammatory diseases and in cancers [5]. PD-1 is one of the most important inhibitory co-receptors expressed predominantly by activated T and B lymphocytes whose expression is sustained by permanent antigenic stimulation [6]. PD-1 belongs to the immunoglobulin CD28/CTLA-4 superfamily, and it has a cytoplasmic domain containing two tyrosine residues located within immunoreceptor tyrosine-based inhibitory and switch motifs (ITIM and ITSM), suggesting that PD-1 represents an important negative regulator of CD4, CD8 and B cell responses to self- and microbial antigens [7, 8]. The molecule modulates the responses of previously activated T and B cells by binding its ligands PD-L1 and PD-L2 that lead to blockade of (1) antigen-specific proliferation, (2) cytokine production as well as (3) cytolytic function [9, 10].

In patients with tonsillitis, chronic and recurrent antigenic stimulation is the main cause for hypertrophy of these organs. It was reported that the expression of PD-1 and PD-1L as a result of persistent antigenic stimulation is increased in patients with chronic viral and bacterial infections; however, their expressions on human B and T lymphocytes in tonsils during chronic infections have never been reported.

## Materials and methods

Hypertrophied tonsils were collected from 57 children undergoing routine tonsillectomy. The average age was 5 years (range 1–18 years). The study group comprised 20 girls and 37 boys. This study was approved by the local ethics committee, and the parents were informed about the use of their children’s tonsils for scientific purposes. This work has been performed in accordance with the ethical standards as laid down in the 1964 Declaration of Helsinki and its later amendments.

### Tonsillar mononuclear cells isolation

Tonsillar mononuclear cells were isolated from tonsillar tissue by enzymatic lyses with 30 mg of collagenase (Biochrom AG, Germany) and 30 mg of DNase (Sigma-Aldrich, Germany). Tonsils obtained by tonsillectomy were cut manually into small pieces and placed in 30 ml of CLT Test medium (Cellular Technology Limited, USA) with enzymes and mixed for 2 h on a magnetic stirrer (300 rpm, temperature 34–37 °C). Next, the cells were washed with PBS (Biochrom AG, Germany). After centrifugation cells were filtered through a cell strainer (40 µm, BD Falcon, USA) and cryopreserved at −80 °C to the time of analysis. The viability was counted.

### Flow cytometry

The surface antigens CD4, CD5, CD8, CD19, PD-1 and PD-1L were stained with respective monoclonal antibodies (anti-CD4 FITC, anti-CD8 PE, anti-PD-1 APC, anti-CD5 FITC, anti-CD5 PE, anti-PD-1L APC, Becton–Dickinson, USA). After incubation 2 × 10^6^ cells were washed twice with PBS and analyzed by flow cytometry for the expression of CD4^+^, CD8^+^, CD19^+^ and CD5^+^/CD19^+^ lymphocytes and expression of PD-1 and PD-1L on CD4^+^, CD8^+^, CD19^+^ and CD5^+^/CD19^+^ lymphocytes. Flow cytometry analysis was performed on a FACSCanto II and analyzed using the FACSDiva software packages (Becton–Dickinson, USA). Each time 100,000 cells were acquired.

### Statistical analysis

Statistical analyses were performed using GraphPad Prism 5 (La Jolla, USA). The *U* Mann–Whitney and one-way ANOVA tests were used to evaluate the differences between subgroups of patients. The correlations of variables were computed with the Spearman rank correlation coefficient. All results are presented as median values with range. Statistical significance was defined as *p* < 0.05.

## Results

Mononuclear cells isolated from tonsils for 57 patients were analyzed using flow cytometry.

### The frequency of B and T lymphocytes in tonsils tissue

We found the median frequency of CD4^+^ T lymphocytes of 14.5 % and the median frequency of CD8^+^ T lymphocytes of 5.5 % in tonsillar tissue. The median percentage of CD19^+^ B cells was 57.5 %, and the frequency of CD5^+^/CD19^+^ B lymphocytes was 2.5 % (Table 1).

**Table 1:** Frequency of B and T lymphocytes in tonsils tissue and in different age groups

	% of CD4^+^ cells	% of CD8^+^ cells	% of CD19^+^ cells	% of CD5^+^/CD19^+^ cells
Median	14.5	5.5	57.5	2.5
Minimum	6.0	2.0	5.7	0.5
Maximum	32.5	12.5	81.5	12.5
Group I (1–5 years)	13.9	5.3	57.6	2.1
Group II (6–10 years)	14.8	6.0	51.9	1.7
Group III (11–18 years)	21.5	6.3	50.0	4.1
Statistical significance				
I vs. II	*p* = 0.2	*p* = 0.37	*p* = 0.1	*p* = 0.5
I vs. III	*p* = 0.02	*p* = 0.03	*p* = 0.02	*p* = 0.08
II vs. III	*p* = 0.13	*p* = 0.48	*p* = 0.26	*p* = 0.14

Next, we analyzed patients in different age groups (group I 1–5 years, group II 6–10 years, group III 11–18 years). The frequency of CD4^+^ and CD8^+^ T lymphocytes increased with the age of patients. The percentage of CD4^+^ was higher in group III than in group I and II (Table 1).

The level of CD8^+^ T lymphocytes was also significantly higher in group III than in groups I and II (Table 1). In contrast to the frequencies of T cells, the percentages of CD19^+^ B lymphocytes decreased with the age of patients. The level of CD19^+^ lymphocytes was higher in group I than in groups II and III (Table 1). Interestingly, the level of CD5^+^/CD19^+^ B lymphocytes was higher in older children than in younger (Table 1).

### The protein expression of PD-1 and PD-1L on T lymphocytes

**Fig. 1:**
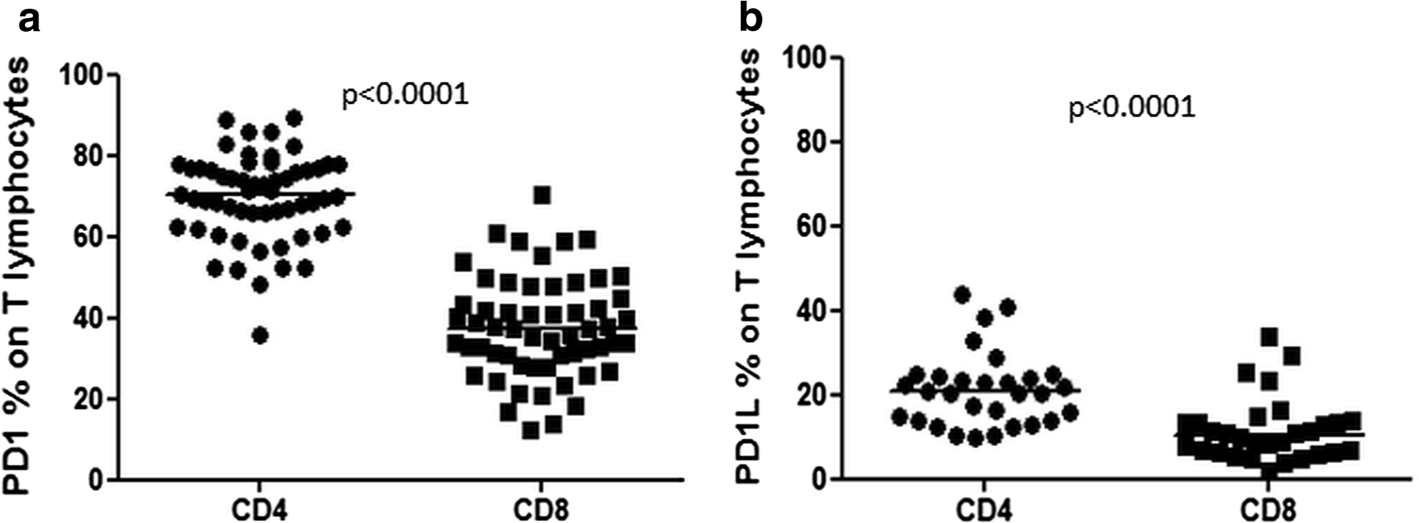
Expression of PD-1 on protein level. **a** Flow cytometric analysis of PD-1 expression on CD4^+^ and CD8^+^ T lymphocytes showed significantly higher expression of PD-1 on CD4^+^ T cells (70 vs. 37 %, *p* < 0.0001). **b** Expression of PD-1L on CD4^+^ was significantly higher than on CD8^+^ T cells (20.7 vs. 10.3 %, *p* < 0.0001)

The expression of PD-1 on CD4^+^ T lymphocytes was significantly higher than on CD8^+^ lymphocytes [median of 70.0 % range 35.8–89.2 % vs. median of 37.0 % range 12.3–70.3 % (*p* < 0.0001)] (Fig. 1a). Also the PD-1L expression on CD4^+^ T lymphocytes was significantly higher than on CD8^+^ T cells [median of 20.0 %, range 9.5–43.5 % vs. median of 10.0 %, range 2.0–33.5 % (*p* < 0.0001)] (Fig. 1b).

We observed no difference in the expression of PD-1 on CD4^+^ in different age groups I–III (Table 2). Similarly, there was no statistically significant difference in PD-1 expression on CD8^+^ lymphocytes according to patients age (Table 2). We did not observe significant differences in PD-1L expression on CD4^+^ lymphocytes and CD8^+^ lymphocytes according to patients age (Table 2).

**Table 2:** Protein expression of PD-1 and PD-1L on T lymphocytes according to patient’s age

	% of PD-1 on CD4^+^ cells	% of PD-1L on CD4^+^ cells	% of PD-1 on CD8^+^ cells	% of PD-1L on CD8^+^ cells
Group I (1–5 years)	71.0	21.3	38.0	10.6
Group II (6–10 years)	70.0	15.4	37.5	7.16
Group III (11–18 years)	52.0	16.7	34.0	13.5
Statistical significance				
I vs. II	*p* = 0.37	*p* = 0.18	*p* = 0.94	*p* = 0.5
I vs. III	*p* = 0.15	*p* = 0.22	*p* = 0.31	*p* = 0.49
II vs. III	*p* = 0.3	*p* = 1.0	*p* = 0.48	*p* = 0.18

Since we earlier reported the expression of PD-1 and its ligand PD-1L on the same chronic lymphocytic leukemia (CLL) cell and could also find a correlation of their expression levels, we analyzed association of PD-1 and PD-1L expression on CD4^+^ and CD8^+^ T cells from tonsils. No correlation between percentages of CD4^+^ T cells expressing PD-1 and PD-1L (*r* = 0.02, *p* = 0.9) nor between CD8^+^ T cells expressing PD-1 and PD-1L was observed (*r* = 0.3, *p* = 0.12).

### The protein expression of PD-1 and PD-1L on B lymphocytes

**Fig. 2:**
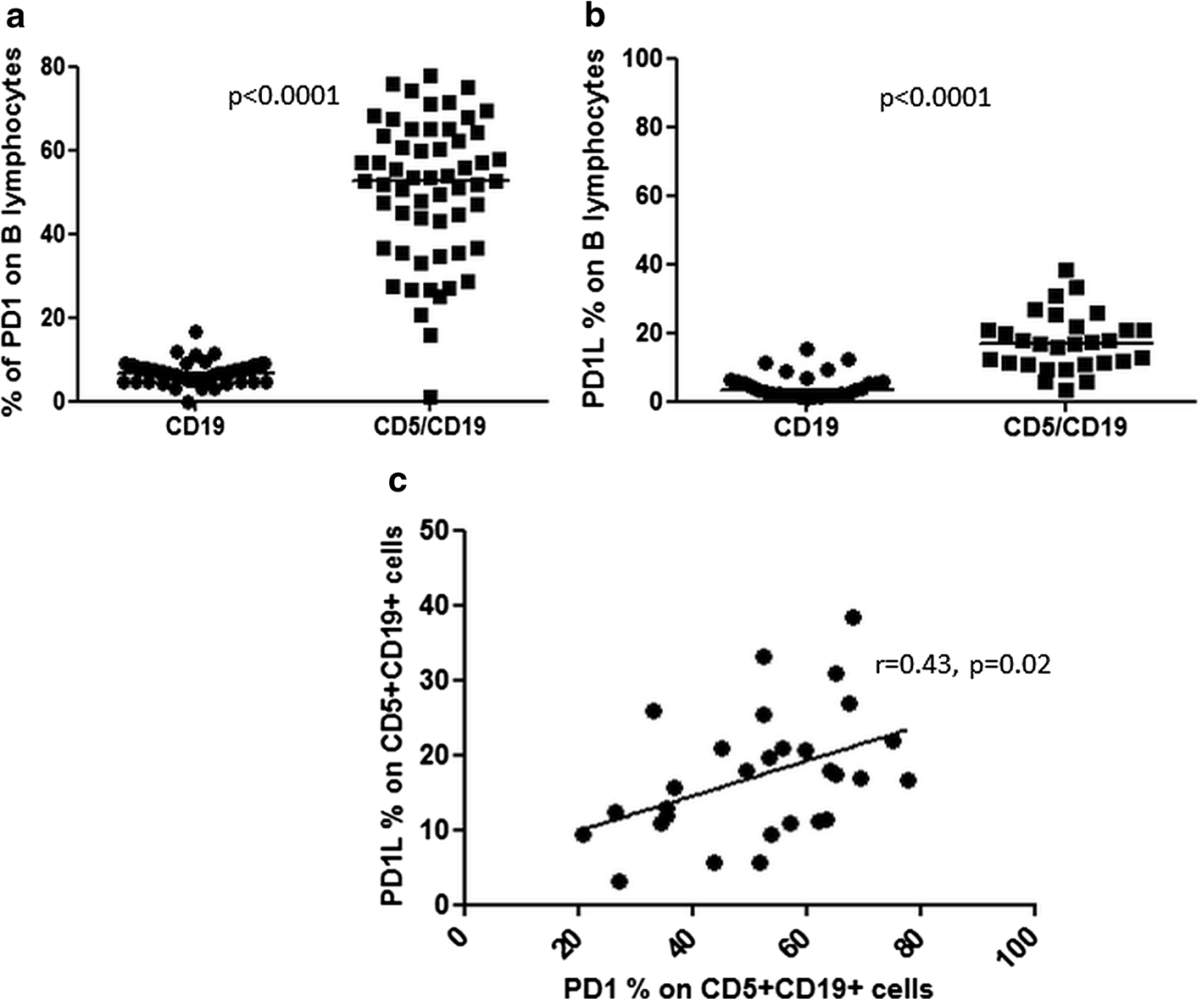
Expression of PD-1 on subpopulation of B lymphocytes. **a** Median PD-1 expression of PD-1 on CD5^+^ CD19^+^ was significantly higher than on CD19^+^ B lymphocytes (52.5 vs. 6.5 %, *p* < 0.0001). **b** Expression of PD-1L was significantly higher on CD5^+^CD19^+^ B cells than on CD19^+^ B lymphocytes (16.8 vs. 3.1 %, *p* < 0.0001). **c** There was a correlation of PD-1 and PD-1L expressions on CD5^+^CD19^+^ cells (*r* = 0.43, *p* = 0.026)

The expression of PD-1 and PD-1L was analyzed in two types of B cells: CD5^+^CD19^+^ B lymphocytes and CD5^−^CD19^+^ B lymphocytes. We observed that the expression of PD-1 on CD5^+^CD19^+^ lymphocytes was significantly higher as compared to PD-1 expression on CD19^+^ lymphocytes (median 52.5 %, range 0.9–78.0 % vs. median 6.5 %, range 0.0–16.5 % (*p* < 0.0001; Fig. 2a). Similarly, the expression of PD-1L on CD5^+^CD19^+^ cells was also significantly higher than on CD19^+^ lymphocytes (median 16.5 %, range 3.05–38.5 % vs. median 3.5 %, range 0.5–15.5 % (*p* < 0.0001; Fig. 2b).

When we analyzed the expression of PD-1 on CD5^+^CD19^+^ cells according to patients age, we observed that the expression of PD-1 is reduced in older children (Table 3), but there was no difference in PD-1L expression. No difference in PD-1 expression on CD19^+^ cells according to patients age was found (Table 3). There was also no difference in PD-1L expression on CD19^+^ cells according to patients age.

**Table 3:** Protein expression of PD-1 and PD-1L on B lymphocytes according to patient’s age

	% of PD-1 on CD19^+^ cells	% of PD-1L on CD19^+^ cells	% of PD-1 on CD5^+^/D19^+^ cells	% of PD-1L on CD5^+^/D19^+^ cells
Group I (1–5 years)	6.9	16.1	57.0	7.0
Group II (6–10 years)	6.8	19.1	51.0	6.5
Group III (11–18 years)	5.0	16.5	27.0	5.0
Statistical significance				
I vs. II	*p* = 0.41	*p* = 0.25	*p* = 0.15	*p* = 0.29
I vs. III	*p* = 0.28	*p* = 0.75	*p* = 0.0046	*p* = 0.76
II vs. III	*p* = 0.43	*p* = 0.75	*p* = 0.004	*p* = 0.72

Interestingly, similarly to our observations in CLL, there was a correlation of PD-1 and PD-1L expressions on CD5^+^CD19^+^ cells (*r* = 0.43, *p* = 0.026; Fig. 2c).

## Discussion

The main objective of this study was to characterize the expression of PD-1 and PD-1L on B and T lymphocytes as well as the frequency of B and T cells in tonsillar tissue during chronic antigenic stimulation accompanying chronic or recurrent tonsillitis. The tonsillitis in most of cases is a result of viral pharyngitis, and approximately 15 % of the cases are evoked by bacterial infection with β-hemolytic streptococci [1]. The tonsillectomy is a routine procedure to treat hypertrophied tonsils, and it is one of the most often performed surgeries during childhood [11].

We found that CD19^+^ B cells were most prevalent subpopulation of lymphocytes in tonsillar tissue. A smaller percentage of tonsillar lymphocytes were T cells with a higher level of CD4^+^ cells than CD8^+^ lymphocytes. Approximately 3 % of the lymphocytes were B1a cells with co-expression of CD5 and CD19. In line with similar distribution pattern of lymphocytes, Graeme-Cook et al. [12] reported that while CD8^+^ cells present diffused localization, CD4^+^ were associated with B cells within the crypt epithelium.

CD5^+^CD19^+^ B1a cells have been found in healthy individuals, in patients who suffer from autoimmune disorders but also in patients with CLL; notably in both situations CD5 expression on CD19 B cells could reflect tolerance mechanisms inhibiting BCR signaling [13, 14].

The population of CD5^+^CD19^+^ B cells is maintained by self-renewal for life with increased risk of dysregulated growth and progression to CLL during aging. Accordingly, Dono et al. [4] reported that CD5^+^ B cells show increased tendency to malignant transformation. Our results showed that the frequency of CD5^+^CD19^+^ B lymphocytes was higher in older patients. Thus, one could speculate that potential reservoir of CD5^+^CD19^+^ cells which could later transform to CLL is lymph organs including tonsils; however, at the moment of analysis no sign of malignant transformation of tonsils was observed irrespective of CD5^+^CD19^+^ levels. Interestingly, CD5^+^CD19^+^ found in tonsils were characterized by the expression of PD-1 and PD-1L, which are also associated with inhibition of the function of B cells.

The population of CD5^+^CD19^+^ B cells that were evaluated in current manuscript is heterogeneous and could include B1a cells as well as B regulatory cells (Bregs). Bregs exhibit immunosuppressive functions via diverse regulatory mechanisms such as Fas/FasL pathway, secretion of IL-10, TGF-β or granzyme B production. IL-10-producing Bregs appear during chronic inflammation (such as tonsillitis) and suppress their progression by downregulating inflammatory cascades associated with IL-1 upregulation. Thereby, Bregs producing IL-10 could act as inhibitory cells during immune-mediated inflammation [15]. Recently, PD-1L^+^ Bregs subpopulation was described. PD-L1^hi^ B cells, interacting with CD4^+^CXCR5^+^PD-1^+^ T follicular helper (Tfh) cells, can limit both memory B cell development and plasma cell differentiation [16]. The enhanced expression of PD-1 on B lymphocytes during inflammation was demonstrated by Nicholas et al. [17] in HIV-infected individuals. In patients with HIV infection, Bregs were shown to suppress HIV-1-specific CD8^+^ T cell responses via secretion of IL-10 and expression of PD-L1 [16]. Blockade of IL-10R and PD-1/PD-L1 pathway showed that Bregs can inhibit antigen presentation and CD4^+^ T cells proliferation and can impair anti-HIV cytotoxic T lymphocyte functions in those patients [18]. The expression of PD-1 on peripheral blood CD5^+^CD19^+^ cells was reported by us, recently [19]. We found higher expression of PD-1 in CLL patients on both mRNA and protein level when compared to control B cells. The correlation of the MFI (mean fluorescence intensity) of CLL cells expressing PD-1 and PD-1L was also observed. This correlation might indicate simultaneous activation of the signaling pathway of PD-1/PD-L1, which could be a potential mechanism for tumor escape from immunosurveillance. In current study, high expression of PD-1L and correlation between the expression of PD-1 and PD-1L on CD5^+^CD19^+^ B cells were also observed. Interestingly, this phenomenon seems to be restricted only to this subpopulation of lymphocytes. No correlations between PD-1 and PD-1L were observed on other lymphocytes populations. The interaction of PD-1 with its ligand PD-L1 in non-lymphoid tissues and on dendritic cells allows regulation of potentially auto-reactive lymphocytes. PD-L1 is expressed constitutively by non-lymphoid, parenchymal organs such as the heart, placenta and skeletal muscle. Up-regulated PD-L1 expression was described in several human cancer types [20–22]. Increased expression of PD-1 and PD-1L on T and B lymphocytes which we observed in tonsils may result from continuous antigen stimulation. To our knowledge, there are no data about the expression of PD-1L on tonsillar B and T cells and its functional consequences; however, correlation of expression between molecule and its ligand observed on CD5^+^CD19^+^ might point to enhanced PD-1/PD-1L signaling.

In this work, we observed the highest protein expression of PD-1 on CD4^+^ T lymphocytes. Similarly, Nicholas et al. [17] reported high expression of PD-1 on CD4+ T cells in peripheral blood of patients infected with HIV. PD-1 expression was also significantly higher in CD4^+^ than in CD8^+^ T lymphocytes and CD19^+^ B lymphocytes.

Chronic and recurrent infections such as HBV infection and CMV infection, but also malignant tumors, are characterized by continuous activation of immunocompetent cells which in a time present reduced ability to secrete effector cytokines and have upregulated the expression of inhibitory receptors such as PD-1. Here, we observed that also chronic and recurrent infections that cause tonsillitis lead to increased expression of PD-1 and PD-1L on B and T lymphocytes of tonsils. The continuous expression of PD-1 molecules defines terminally differentiated “exhausted” T cells. Interestingly, Speiser et al. [23] proposed that this “exhausted” phenotype results from differentiation process in which T cells set permanently their effector ability to the needs of chronic infection. They also suggested that this phenotype might be optimal to protect tissue against damage caused by chronic inflammation but still maintain a critical level of pathogen control. Moreover, Lyford-Pike et al. [24] evaluated the expression of PD-1 and PD-1L in cancerous (HPV-associated head and neck cancer) and noncancerous tonsils. They reported high expression of PD-1 on CD4^+^ and CD8^+^ T cells in chronic tonsillitis patients, but this expression was significantly lower than in cancerous tonsils. In functional studies, Lyford-Pike et al. [21] did not observed significant differences in the functional capacity of CD8^+^PD1^+^ versus CD8^+^PD-1^−^ T cells in the peripheral blood and tonsils of chronic tonsillitis patients. In contrast to tonsillitis in HPV-associated head and neck squamous cell carcinoma patients, CD8^+^PD1^+^ tumor-infiltrating lymphocytes showed decreased ability to produce effector cytokines. We could speculate that tonsillar lymphocytes which are characterized by high expression of PD-1 and PD-1L are in fact adapted to chronic antigenic stimulation. Interestingly, Sada-Ovalle et al. [8] reported in their study that CD4^+^ and CD8^+^ tonsillar T lymphocytes obtained by tonsillectomy from patients with tonsillitis are fully functional. They proved that tonsillar CD4^+^ and CD8^+^ T cells are able to respond to stimulation, activate and then differentiate into antigen-specific and effector cells.

Functional studies have shown that PD-1^+^CD8^+^ T lymphocytes in tumor tissue are dysfunctional, while these cells in tumor-free lymph nodes are functional [25]. These results suggest that other molecules and inhibitory pathways might be involved in T lymphocyte exhaustion in cancer patients and patients with chronic or recurrent infections such as tonsillitis. However, gene profiling studies have demonstrated that molecular profile of exhausted T cells in malignancy is similar to the one observed in chronic viral infections [5]. We could speculate that high expression of PD-1 and its ligand PD-1L on T and B cells found in patients with tonsillitis does not inhibit their function in contrast to PD-1 expression on T cells of cancer patients and local immune dysfunction may be associated with fibrosis of the tonsillar epithelium.

In summary, in this study we observed that chronic and recurrent infections that cause tonsillitis lead to increased expression of PD-1 and PD-1L on B and T lymphocytes of tonsils. High expression of PD-1 and PD-1L could represent hallmark of immune system adaptation to chronic antigenic exposition in patients with tonsillitis.
